# Impact of health expenditure on universal health coverage (UHC) (composite index): Global evidence

**DOI:** 10.34172/hpp.025.43192

**Published:** 2025-11-04

**Authors:** Mohsin Raza Khan, Khalil Ullah Mohammad, Muhammad Saad Rabbani

**Affiliations:** ^1^Department of Business, Bahria Business School, Bahria University, Islamabad, Pakistan; ^2^Department of Management Sciences, Bahria Business School, Bahria University, Islamabad, Pakistan

**Keywords:** Health expenditure, Infectious diseases, Non-communicable diseases, Public health expenditure, Universal health coverage, Women’s health, Domestic health expenditure, UHC composite index, Fixed effect model

## Abstract

**Background:**

In recent years, global commitments to achieving universal health coverage (UHC) have emphasized the critical importance of public health funding. This study aims to explore the relationship between public health expenditure (PHE) and UHC.

**Methods:**

The study is based on Grossman’s health production model, which posits that health is a commodity requiring investment. Data for this analysis was sourced from the World Bank’s World Development Indicators and the World Health Organization’s Global Health Expenditure Database, covering 169 countries over a 22-year period from 2000 to 2022. Both fixed and random effect panel regressions were conducted using STATA for the analysis.

**Results:**

The findings indicate that per capita domestic health expenditure significantly impacts health outcomes (0.068, 95% CI: 0.0336,0.1038), especially in combating infectious diseases (0.2543, 95% CI: 0.1552,0.3533). Additionally, higher education completion rates are linked to better health outcomes (0.0020, 95% CI: 0.0008,0.0032). The results also suggest that an aging population may require increased resources for managing non-communicable diseases (0.0184%, 95% CI: 0.0121,0.0246) and Service Capacity an access (0.0140, 95% CI: 0.0022,0.0259). Furthermore, higher life expectancy at birth strongly correlates with improved health outcomes across various sectors, marking it as a robust indicator of overall health (0.0339, 95% CI: 0.0226,0.0453). The findings indicate that per capita domestic health expenditure significantly impacts health outcomes, especially in combating infectious diseases. Additionally, higher education completion rates are linked to better health outcomes. The results also suggest that an aging population may require increased resources for managing non-communicable diseases and service capacity an access. Furthermore, higher life expectancy at birth strongly correlates with improved health outcomes across various sectors, marking it as a robust indicator of overall health.

**Conclusion:**

Our analysis using fixed effect models revealed significant factors affecting health outcomes in reproductive, maternal, newborn, and child health (RMNCH); infectious diseases (ID); non-communicable diseases (NCD); and service capacity and access (SC). Strategic health investments and policies in areas like infectious diseases, where funding directly improves health outcomes, could greatly enhance these results. Our data strongly supports increasing and strategically allocating health expenditure to maximize impact.

## Introduction

 Universal health coverage (UHC) focuses on ensuring that everyone can access essential health services without suffering financial difficulties, regardless of when or where they need them.^1^ This includes a broad range of services, from preventive care to curative treatments, immunizations, and other vital healthcare services. UHC also plays a pivotal role in promoting economic development by improving population health, boosting productivity, and reducing the financial burden of disease on individuals and families.^2^

 The importance of achieving UHC has gained increased global attention over the past decade. A significant milestone was the global commitment made in Geneva in 2012, where leaders worldwide endorsed UHC as a key goal and agreed to achieve it by 2030.^3,4^ This commitment aligns with Sustainable Development Goal (SDG) 3.8, which emphasizes UHC as a critical target for global health equity. Additionally, international collaborations, such as the Global Alliance for Chronic Diseases and the European Union’s Health Programme, highlight joint research and cross-border healthcare initiatives aimed at addressing health gaps.^5^ These partnerships strengthen the global commitment to achieving UHC and underscore the importance of shared efforts in fostering equitable access to healthcare.

 Despite its significance, many countries face substantial challenges in progressing toward UHC. Insufficient healthcare financing is a primary obstacle, as governments struggle to allocate adequate resources to establish and sustain comprehensive coverage. Furthermore, a heavy reliance on out-of-pocket (OOP) expenditure exacerbates inequities, leaving many without access to necessary care.^3^ Global health emergencies, such as the COVID-19 pandemic, further strain healthcare systems, diverting resources from long-term UHC initiatives and exposing infrastructural weaknesses.^6^ In this context, effective utilization of public funds emerges as a potential solution for advancing UHC.^7^

 Research consistently highlights the critical role of public health expenditure (PHE) in achieving UHC. Sustainable financing through public sources ensures financial stability, predictability, and equity in healthcare provision.^8^ Public funding reduces the financial burden on individuals and ensures consistent access to essential services. Recognizing this, the present study explores the relationship between PHE and UHC to better understand the dynamics of healthcare financing. Specifically, the study investigates whether public investments in primary healthcare (PHC) contribute to achieving UHC and examines the correlation between a country’s economic status and its UHC score, with a focus on higher-income countries.

 This study adopts a distinctive methodology by disaggregating UHC into four composite indices: reproductive, maternal, newborn, and child health (RMNCH); infectious diseases (ID); non-communicable diseases (NCD); and service capacity and access (SC). By analyzing these components individually, the study aims to provide deeper insights into the specific impacts of public expenditure across various healthcare sectors. This nuanced approach offers valuable guidance for policymakers seeking to optimize resource allocation and enhance the effectiveness of public spending in achieving UHC objectives.

 The findings of this study have far-reaching implications for multiple stakeholders. Governments can utilize these insights to refine policy decisions and allocate resources more effectively. Donors can make informed funding decisions to support impactful initiatives, while the general public benefits from improved healthcare services resulting from evidence-based policymaking.

 Investing in health is widely recognized as a fundamental strategy for improving health outcomes. A growing body of research underscores the significant impact of health expenditures on population well-being. Studies consistently reveal a positive correlation between higher levels of investment and improved health indicators, emphasizing the importance of financial resources in enhancing collective health. Public health spending, in particular, plays a critical role in ensuring accessible, high-quality healthcare services. It serves as a foundational pillar for UHC implementation by enabling governments to manage and deliver essential services effectively.

 However, empirical studies highlight the complexities underlying the relationship between health expenditure and UHC. While some research demonstrates a positive association between increased spending and greater healthcare utilization, other studies point to inefficiencies, unequal resource distribution, and socio-economic disparities as barriers to achieving equitable health outcomes. For example, regional differences in healthcare infrastructure and socio-economic challenges necessitate tailored approaches, as a uniform strategy may not yield consistent results across diverse contexts.

 Theoretical frameworks such as the Human Capital Model and the Health Production Function provide additional perspectives on the relationship between health expenditure and UHC.^9,10^ The Human Capital Model views health as an investment, emphasizing how improved health enhances productivity and economic growth. This perspective highlights the broader societal benefits of increased health spending. Conversely, the Health Production Function approach conceptualizes health as a “commodity” produced by healthcare systems,^11^ emphasizing the importance of efficient resource allocation and system management in maximizing health outcomes.^12^ These frameworks underscore the need for comprehensive strategies that address socio-economic determinants of health, such as poverty and inequality, alongside increased financial investments.

 Moreover, the effectiveness of public health spending is influenced by factors such as income levels, governance quality, and the composition of healthcare expenditures. Cross-country analyses reveal a complex interplay between these variables. While higher income levels often correlate with better health outcomes, public spending alone does not guarantee success. For instance, a study from Sub-Saharan Africa found that investments in intermediate healthcare goals, such as immunization coverage, yielded greater success than broader objectives like reducing mortality rates. This suggests that targeted spending on specific healthcare priorities can achieve more immediate and measurable improvements.

 The role of governance and institutional quality further complicates the relationship between public spending and health outcomes. Studies indicate that countries with robust governance frameworks and efficient healthcare systems are better positioned to translate financial investments into tangible health improvements. Conversely, weak governance and systemic inefficiencies can undermine the impact of increased spending, leading to suboptimal outcomes. Addressing these challenges requires a holistic approach that integrates financial investment with institutional strengthening and policy reforms.

 Despite extensive research, gaps remain in understanding the nuanced relationship between health expenditure and UHC. Existing studies often produce mixed results, making it difficult to draw definitive conclusions. Additionally, many studies fail to distinguish between public and private sources of health financing, which may have differing effects on health outcomes. These limitations highlight the need for more granular analyses to inform evidence-based policymaking.

 The current study aims to address these gaps by employing a refined methodology that disaggregates UHC into its constituent components. This approach allows for a detailed examination of how public health investments impact specific healthcare sectors, providing a comprehensive understanding of the dynamics at play. By focusing on the interplay between PHE, economic status, and healthcare outcomes, the study seeks to generate actionable insights that can inform policies and strategies for advancing UHC.

## Material and Methods

###  Study design and population

 This study adopts a quantitative panel data analysis design to study the impact of government health expenditure on health outcomes of countries. Using a sample of 169 countries data a log-log fixed effect model is employed controlling for unobserved time invariant heterogeneity across countries. We estimate the impact of health expenditure on UHC index, heterogeneous impact of health expenditure across income levels and nonlinearity in the relationship using three econometric models. Finally, we decompose the UHC index into its composite factors to see how health expenditure impacts them.

 Grossman proposed a model of demand for health capital which we ground our econometric design.^13^ They suggested that good health is a commodity that is a form of durable capital, positively associated with investment and negatively associated with age. Besides investment (price of medical care) there are various other factors that impact the capital stock of health (shadow prices).


(1) $$H=F\left(Y,S,E,D\right)$$

The model suggests that health outcome *H* is dependent on health expenditure *Y*, social factors *S*, education and population characteristics, Environmental factors *E*, availability of fresh drinking water and sanitation services, and the availability of health utilization services *D*, measles immunization etc. The scaler form of equation [1] is given in the equation [2].


(2) $$H=\Omega \;\prod y_{i}^{\alpha_{i}}\;\prod s_{i}^{\beta_{m}}\;\prod e_{i}^{\lambda_{i}}\;\prod D_{i}^{\gamma_{i}}$$

Where Ω is the initial health stock as per the Grossman model, elasticities of the model are represented by α_i_,β_m_,λ_i_ γ_i_ and ∏ represent the population density function. Taking the logarithm of Equation [2] results in a linearized equation [3].


(3) $$ln\;H=ln\;\Omega +\sum \alpha_{i}\left(lny_{i}\right)\;+\sum \beta_{m}\left(ln\;s_{i}\right)\;+\sum \lambda_{i}\left(lne_{i}\right)+\sum \gamma_{i}\left(lnD_{i}\right)$$

In this equation [3] the ∑ operator summation of all the factors within these categories. The equations represent the log of health expenditure *(ln y)*, social factors *(ln s)*, environmental *(ln e)*, and health services utilization factors *(ln D)*.

Equation [4a] estimates the main model to analyze how government expenditure impacts the UHC index. In extending the literature further we estimate [4b], to see if economic development levels proxied by income classifications matter. Equation [4c] includes a quadratic term to explore the functional form of the relationship.


(4a) $$\begin{array}{l} UHC_{tc}=\alpha_{0}+\;\alpha_{1}lnExpend_{tc}+\beta_{1}Educ_{tc}+\\ \beta_{2}OldPopulation_{tc}+\beta_{3}YoungPopulation_{ct}+\lambda_{1}WaterAvailability_{ct}\\ +\lambda_{2}SanitationService_{ct}++\gamma_{1}Measles_{tc}+\gamma_{2}Lifexpectancy_{tc}+\mu_{tc} \end{array}$$

(4b) $$\begin{array}{l} UHC_{tc}=\alpha_{0}+\;\alpha_{1}lnExpend_{tc}+\beta_{1}Educ_{tc}+\beta_{2}OldPopulation_{tc}\\ +\beta_{3}YoungPopulation_{tc}+\lambda_{1}WaterAvailability_{tc}+\lambda_{2}SanitationService_{tc}\\ +\gamma_{1}Measles_{tc}+\gamma_{2}Lifexpectancy_{tc}+\delta_{z}IncomeClassification\;x\;lnExpend{\;}_{tc}+\mu_{tc} \end{array}$$

(4c) $$\begin{array}{l} UHC_{tc}=\alpha_{0}+\;\alpha_{1}lnExpend_{tc}+\alpha_{1}lnExpend_{tc}{}^{2}+\beta_{1}Educ_{tc}+\beta_{2}OldPopulation_{tc}\\ +\beta_{3}YoungPopulation_{ict}+\lambda_{1}WaterAvailability_{ict}+\lambda_{2}SanitationService_{ict}\\ +\gamma_{1}Measles_{tc}+\gamma_{2}Lifexpectancy_{tc}+\delta_{z}IncomeClassification\;x\;lnExpend{\;}_{tc}+\mu_{tc} \end{array}$$

UHC index composite factors include RMNCH, NCD, ID and Service Capacity and we use them to estimate how health expenditure impacts these factors individually u


(5) $$\begin{array}{l} RMNCH_{tc}=\alpha_{0}+\;\alpha_{1}lnExpend_{tc}+\beta_{1}Educ_{tc}+\beta_{2}OldPopulation_{tc}\\ +\beta_{3}YoungPopulation_{tc}+\lambda_{1}WaterAvailability_{tc}+\\ \lambda_{2}SanitationService_{tc}+\gamma_{1}Measles_{tc}+\gamma_{2}Lifexpectancy_{tc}+\mu_{tc} \end{array}$$

(6) $$\begin{array}{l} NCD_{tc}=\alpha_{0}+\;\alpha_{1}lnExpend_{tc}+\beta_{1}Educ_{tc}+\beta_{2}OldPopulation_{tc}\\ +\beta_{3}YoungPopulation_{tc}+\lambda_{1}WaterAvailability_{tc}+\\ \lambda_{2}SanitationService_{tc}+\gamma_{1}Measles_{tc}+\gamma_{2}Lifexpectancy_{tc}+\mu_{tc} \end{array}$$

(7) $$\begin{array}{l} ID_{tc}=\alpha_{0}+\;\alpha_{1}lnExpend_{tc}+\beta_{1}Educ_{tc}+\beta_{2}OldPopulation_{tc}\\ +\beta_{3}YoungPopulation_{tc}+\lambda_{1}WaterAvailability_{tc}\\ +\lambda_{2}SanitationService_{tc}+\gamma_{1}Measles_{tc}+\gamma_{2}Lifexpectancy_{tc}+\mu_{tc} \end{array}$$

(8) $$\begin{array}{l} ServiceCap_{tc}=\alpha_{0}+\;\alpha_{1}lnExpend_{tc}+\beta_{1}Educ_{tc}+\\ \beta_{2}OldPopulation_{tc}+\beta_{3}YoungPopulation_{tc}+\lambda_{1}WaterAvailability_{tc}\\ +\lambda_{2}SanitationService_{tc}+\gamma_{1}Measles_{tc}+\gamma_{2}Lifexpectancy_{tc}+\mu_{tc} \end{array}$$

###  Data collection

 The data comprises country-level annual observations on health outcomes, health expenditure, education, demographics, environment, and disease incidence.Health outcomes are proxied in the study using UHC index which is calculated at the country level. Out of 266 countries globally, WHO has calculated the index for 169 countries all of which was used from 2000 to 2022. During this period the index was computed 7 times resulting dataset in an unbalanced panel dataset. We do not impute the missing values by interpolation to avoid data smoothing. However, using all 169 countries covering high-, middle- and low-income countries allows for generalization of our findings (see Supplementary file 1). ‘lnExpend’ is log of Domestic General Health Expenditure (DGG) per capita (current US$) proxying health expenditure. For social factors the estimation model includes the log of Primary completion rate (% of relevant age group), demographic factors the log of (Population aged 65 and above (% of total population), and Population aged between 15 and 64 (% of total population). For the environmental factors two proxies are used. These are People using at least basic drinking water services (% of population) and People using at least basic sanitation services (% of population). Finally, for health services utilizations factors we use the log of number of reported cases of measles. The data is taken from two major sources: The World Bank’s World Development Indicators (WDI) and the World Health Organization’s Global Health Expenditure Database (see Supplementary file 2 for sources of data).

###  Data analysis

 The data underwent analysis in STATA 15 software. Our analysis method is informed by the Hausman test. The *P *value of the Hausman test was less than 1% suggesting the use of fixed effect estimation (Supplementary file 3). By employing log log fixed effect model, we control for country level confounding variables. The Cook- Weisberg test rejects the constant variance hypothesis, and we report Whites robust standard errors in our results to correct for heteroskedasticity (Supplementary file 4). We also test for multicollinearity using the variance inflation factor and find the mean average variance inflation factor is 4.006 suggesting no issues of multicollinearity (see Supplementary file 5). The data is assumption for normality is satisfied due to the asymptotic properties of the least squared estimator. For final robustness of our findings, we report the random effects estimation in Supplementary file 6.

## Results

 The study focused on four health outcomes that were composites of the UHC index: RMNCH, ID, NCD, and SC (Table 1). Many researchers use the UHC index, life expectancy rate, and infant mortality rate to represent a population’s health condition.

**Table 1:** Descriptive Statistics of Key Variables (2000–2022)

**Variables**	**Mean**	**SD**	**Minimum**	**Maximum**	**Source**
UHC Index	3.999	0.385	2.565	4.489	WDI
RMNCH	4.222	0.283	2.773	4.564	WHO
NCD	4.014	0.231	2.485	4.443	WHO
ID	3.767	0.703	1.386	4.585	WHO
SC	3.993	0.616	2.079	4.605	WHO
Measles (number of reported cases)	4.875	2.875	0.000	12.716	WHO
Domestic general health expenditure per capita (current US$)	4.505	2.042	-1.886	8.969	WDI
Primary completion rate, total (% of relevant age group)	88.067	19.242	16.564	134.546	WDI
Population aged 65 and above (% of total population)	7.394	5.437	0.172	24.054	WDI
Population aged between 15 and 64 (% of total population)	62.488	6.924	47.287	86.079	WDI
People using at least basic drinking water services (% of population)	4.399	0.27	2.928	4.605	WDI
People using at least basic sanitation services (% of population)	4.091	0.672	1.027	4.605	WDI
GDP Constant (US$)	3.172	0.097	2.843	3.423	WDI

Abbreviations: UHC: Universal Health Coverage; RMNCH: Reproductive, Maternal, Newborn, and Child Health; GDP: Gross Domestic Product; WDI: World Development Indicators; WHO: World Health Organization.

 A total of 7 models were estimated 4 a-c, 5, 6, 7 and 8, the results of which were reported in Table 2 and Table 3. Table 2 presents the results of equations 4a, 4b, and 4c, each testing a different hypothesis regarding UHC. Hypothesis 1 examines the impact of health expenditure on UHC, hypothesis 2 explores how the income level of countries affects UHC, and hypothesis 3 investigates whether the relationship between health expenditure and UHC is linear or nonlinear. The interpretations of these results are as follows:

**Table 2:** Fixed effects estimation results: impact of health expenditure on UHC and income-level interaction

	**Hypothesis 1**	**Hypothesis 2**	** Nonlinearity**
	**Coefficient/robust standard errors **	**Coefficient/robust standard errors**	**Coefficient/robust standard errors**
DGG health expenditure per capita	0.0687***	0.0705***	0.1165**
	(0.0178)	(0.0169)	(0.0372)
Primary completion rate	0.0020**	0.0020**	0.0018**
	(0.0006)	(0.0006)	(0.0006)
Population ages 65 and above (% of total population)	0.0027	0.0060	0.0104**
	(0.0051)	(0.0046)	(0.0047)
Population ages 15-64 (% of total population)	0.0069*	0.0068**	0.0083**
	(0.0035)	(0.0034)	(0.0034)
Basic drinking water services (% of population)	0.0044	0.0042	0.0036
	(0.0027)	(0.0026)	(0.0026)
Basic sanitation services (% of population)	0.0047**	0.0044**	0.0040**
	(0.0015)	(0.0015)	(0.0015)
Measles (number of reported cases)	< -0.0001	< -0.0001	< -0.0001
	( < 0.0001)	( < 0.0001)	( < 0.0001)
Life expectancy at birth, total (years)	0.0339***	0.0341***	0.0344***
	(0.0058)	(0.0059)	(0.0058)
Higher income x domestic expenditure		< -0.000**	< -0.0001
		( < 0.0001)	( < 0.0001)
Lower income x domestic expenditure		0.0013	-0.0002
		(0.0041)	(0.0038)
Lower Mid income x domestic expenditure		< 0.0001	0.0001
		(0.0003)	(0.0003)
(Domestic expenditure)^2^			-0.0062*
			(0.0037)
Constant	-0.0368	-0.0372	-0.1388
	(0.3469)	(0.3531)	(0.3486)
R-squared	0.844	0.846	0.848
Adj. R-squared	0.8421	0.8435	0.8451
Overall R square	0.8501	0.8522	0.8527
No. of observations	696	696	696
No. of groups	169	169	169
Reference dummy is middle income country			

* *P* < 0.1, ** *P* < 0.05, *** *P* < 0.001.

**Table 3:** Fixed effects model estimations for health outcomes by UHC components

	**Decomposition**			
**Variables**	**RMNCH**	**NCD**	**ID**	**SC**
	**Coefficient/robust standard errors **	**Coefficient/robust standard errors **	**Coefficient/robust standard errors **	**Coefficient/robust standard errors **
DGG health expenditure per capita	0.0227	0.0074	0.2543***	-0.0022
	(0.0156)	(0.0119)	(0.0502)	(0.0153)
Primary completion rate	0.0035***	0.0016**	0.0039*	-0.0004
	(0.0006)	(0.0005)	(0.0022)	(0.0009)
Population ages 65 and above (% of total population)	-0.0149**	0.0184***	-0.007	0.0140**
	(0.005)	(0.0032)	(0.0156)	(0.006)
Population ages 15-64 (% of total population)	-0.0053*	0.0028	0.0278**	0.0021
	(0.003)	(0.0018)	(0.0101)	(0.0038)
Basic drinking water services (% of population)	-0.0018	0.0013	0.0112	0.0073**
	(0.0033)	(0.0017)	(0.0073)	(0.0027)
Basic sanitation services (% of population)	0.0031*	0.0035**	0.0113**	0.0008
	-(0.0018)	(0.0016)	(0.0041)	(0.0018)
Measles (number of reported cases)	< 0.0001	< 0.0001	< 0.0001	< 0.0001
	( < 0.0001)	( < 0.0001)	( < 0.0001)	( < 0.0001)
Life expectancy at birth, total (years)	0.0216***	0.0119***	0.0965***	0.0034
	(0.0057)	(0.0029)	(0.017)	(0.0039)
Constant	2.6935***	2.3236***	-8.0286***	2.9034***
	(0.3848)	(0.1748)	(0.9547)	(0.2923)
R-squared	0.596	0.713	0.812	0.201
Adj. R-squared	0.5911	0.7097	0.8098	0.1912
Overall R-square	0.6388	0.2721	0.5719	0.7726
No. of observations	696	696	696	696
No. of groups	169	169	169	169

Note: *** denotes significance at the 1% level, ** denotes significance at the 5% level, * denotes significance at the 10% level.

 Model 5-8 were estimated to check how the selected variables (Health expenditure, education, age sanitation etc.) has an impact on RMNCH, ID, NCD and SC. All these models performed satisfactorily in the Breusch and Pagan Lagrange Multiplier (LM) test. Despite the preference for the GLS-fixed effects indicated by the Hausman test findings, we included both the GLS-fixed (see Table 3) and GLS-random effect estimates (see Supplementary file 6) models for comparison and to ensure robustness checks. However, the data are primarily interpreted and discussed concerning the fixed effect model. Robust standard errors were applied to both fixed and random effect models to address any potential uniformity issues.

Table 3 presents fixed effects estimation results for different health outcomes and variables across four categories: RMNCH, NCD, ID, and SC. Each row represents a different variable and its estimated impact on the respective health outcome category, with significance levels indicated by asterisks. Standard errors are listed below the coefficients in smaller type.

###  Interpretation of descriptive statistics

 The UHC index was derived as the geometric average of two indices, the health service coverage index and the financial risk protection (FP) index, by the joint WHO-World Bank monitoring framework. The SC index, based on the WHO database, combines 14 similar tracer indicators of health services into a single summary index. These indicators focus on RMNCH, ID, NCD and SC.

 Due to data limitations on catastrophic and impoverishing health expenditures, which are required to measure the FP index as recommended in the UHC monitoring framework, the study followed the approach outlined by Jordi et al. and used the complement of OOP payments as a share of current health expenditure (CHE) as a proxy for the FP index. As a result, the UHC index was created by taking a simple geometric average of the SC index from the World Health Organization’s database and the FP index from OOP health spending (i.e., SC X FP). The data sources for all control variables are listed in Supplementary file 1.

Table 1 provides an overview of the mean, standard deviation, minimum, and maximum values for each variable across the period 2000-2022.

 The average UHC index score is 3.999, with a standard deviation of 0.385. This suggests that there is variability in the level of UHC achieved within the examined countries. Scores vary from 2.565 to 4.489. The component indices also demonstrate some variability. The RMNCH score averages 4.222, whereas the NCD and ID indices average 4.014 and 3.767, respectively. SC have a comparable mean value of 3.993. It is worth noting that the ID index has a large standard deviation (0.703), implying that countries’ ability to deal with IDs varies significantly.

 Moreover, measles cases, taken as a measure of infectious disease control, have a concerning average of 4.875 reported cases. This demonstrates the ongoing difficulty of controlling IDs in some countries, with a maximum reported case of 12.716. Domestic general health expenditure per capita (in current US dollars) varies significantly by country. The average spend is $4.505, while the range includes negative values (-1.886) and a high of $8.969.

 Looking at larger demographic and socioeconomic factors, the primary completion rate for the relevant age group is a promising 88%, with a standard deviation of 19.2. However, there is some variation, with some countries reaching completion rates as high as 134.6% and others falling short at 16.6%. The average population aged 65 and up is 7.394%, with a maximum of 24%, indicating an aging population in several countries. The working-age population (15-64 years) accounts for an average of 62.49%, with a standard deviation of 6.924%. Finally, access to basic drinking water and sanitation services has positive averages of 4.399% and 4.091%, respectively, with minor variance between nations.

###  Interpretation of results of equation 4a-c estimations 

 The analysis of equation 4a-c (Fixed Effects Table 2) highlights the relationship between health expenditure per capita and health outcomes across countries of varying income levels. The findings show that while increased health expenditure per capita positively influences health outcomes, the extent of this effect is strongly moderated by a country’s income level. High-income countries exhibit diminishing returns on additional health investments due to already robust healthcare infrastructure, whereas low and lower-middle-income countries do not show a consistent or significant relationship, likely due to inefficiencies or structural barriers.

 Specifically, higher health expenditure per capita has a significant positive effect in improving health outcomes, as seen in both hypotheses. However, the interaction between domestic health expenditure and income levels reveals nuanced effects. In high-income countries, increased domestic expenditure shows diminishing returns, indicated by a significant negative interaction term. For lower and lower-middle-income countries, no significant impact is observed, with only a slight, non-significant positive interaction noted in Hypothesis 1. This suggests that while high-income countries may benefit less from additional expenditure, low-income nations face challenges in translating such investments into improved outcomes due to inefficiencies or other constraints.

###  Interpretation of results of equation 5-8 estimations 

 The equation 5-8 (Fixed Effects Table 3) provides an in-depth exploration of various determinants of health outcomes across different sectors, highlighting the significance of economic, demographic, and infrastructural factors. The study emphasizes the complexity of health dynamics by examining variables such as health expenditure per capita, education levels, population demographics, sanitation access, life expectancy, and disease-specific trends. It identifies areas where targeted interventions, such as improving sanitation or investing in education, can significantly enhance health outcomes, while also noting sectors where certain variables have negligible or inconsistent effects. The detail interpretation of results is as follows.

####  DGG health expenditure per capita

 DGG health expenditure per capita significantly impacts health outcomes, particularly in combating ID. For instance, a 1 unit increase in DGG health expenditure per capita results in a substantial and significant increase of 0.2543 units in outcomes for ID, underscoring the effectiveness of increased health spending in this area. However, the effects on other health sectors are less pronounced and statistically insignificant; a 1 unit increase leads to only a 0.0227 unit increase in RMNCH outcomes and a 0.0074 unit increase in NCD outcomes. Moreover, a similar increase in expenditure results in a negligible decrease of 0.0022 units in Social Care outcomes, which also lacks statistical significance, indicating a minor and slightly negative effect in this sector.

####  Primary completion rate

 The primary completion rate is positively associated with improvements in RMNCH, NCDs, and IDs, suggesting that higher education completion rates contribute to better health outcomes in these areas. Specifically, a 1 percentage point increase in the primary completion rate is linked to a 0.0035 unit increase in RMNCH outcomes, significant at the 1% level, a 0.0016 unit rise in NCD outcomes, significant at the 5% level, and a 0.0039 unit enhancement in ID outcomes, significant at the 10% level. However, the impact on SC outcomes is negligible (-0.0004) and statistically insignificant, indicating little to no effect in this area.

####  Population ages 65 and above (% of total population)

 The percentage of the population aged 65 and above has varied impacts on different health sectors. Specifically, a 1 percentage point increase in this demographic is associated with a decrease of 0.0149 units in RMNCH outcomes, which is statistically significant at the 5% level, indicating a negative impact. Similarly, the same increase results in a decrease of 0.007 units in outcomes for IDs, though this change is not statistically significant. Conversely, the aging population positively affects NCDs and SC; a 1 percentage point increase in the elderly population leads to an increase of 0.0184 units in NCD outcomes, significant at the 1% level, and an increase of 0.0140 units in SC outcomes, significant at the 5% level. These results suggest that an older population may require more resources for managing NCDs and SC, reflecting better outcomes in these areas as the elderly demographic grows.

####  Population ages 15-64 (% of total population)

 Has a mixed impact across categories, with a significant positive correlation with IDs, suggesting that a larger working-age population may increase the transmission of IDs but also potentially improve economic productivity, impacting health care resources.

####  Basic sanitation services (% of population)

 The percentage of the population with access to basic sanitation services demonstrates significant positive impacts on health outcomes, particularly for NCDs and IDs, highlighting the crucial role of sanitation in disease control and general health improvement. Specifically, a 1 percentage point increase in access to basic sanitation services results in a 0.0031 unit increase in RMNCH outcomes, significant at the 10% level. For NCD outcomes, the same increase leads to a 0.0035 unit rise, significant at the 5% level. Additionally, ID outcomes experience a more pronounced improvement of 0.0113 units, also significant at the 5% level. However, the impact on SC outcomes is minimal, with only a 0.0008 unit increase, which is not statistically significant, suggesting a lesser effect in this area.

####  Life expectancy at birth, total (years)

 Higher life expectancy at birth correlates strongly and positively with improved health outcomes across various sectors, indicating it as a robust indicator of overall health. Specifically, each additional year in life expectancy at birth is associated with a 0.0216 unit increase in RMNCH outcomes, significant at the 1% level. The same additional year leads to a 0.0119 unit rise in NCD outcomes, also significant at the 1% level. In the case of ID, each additional year results in a significant increase of 0.0965 units, underscoring a substantial positive impact. SC outcomes also benefit, with each additional year contributing to a 0.0034 unit increase, which is statistically significant, though minor. These findings demonstrate the critical role of life expectancy as a determinant of health across various medical domains.

####  Measles (number of reported cases)

 Interestingly, this variable has no significant effect in any category, suggesting that measles incidence may be low enough not to impact the overall health outcomes significantly or other factors may be controlling its impact.

## Discussion

 The results of our fixed effects model provide insightful revelations into the drivers of health outcomes across four distinct categories: RMNCH, NCDs, IDs, and SC. Several key findings emerge from our analysis that both align with and extend the existing literature on health economics and public health.

 The study found that increased per capita health expenditure significantly improves health outcomes in IDs, while its impact on other sectors such as RMNCH and NCDs remains minimal. The analysis indicates that increased per capita health expenditure significantly enhances outcomes in IDs (*P* < 0.01), which is consistent with literature suggesting that higher health spending can improve access to necessary services and reduce disease incidence.^14^ This effect is particularly pronounced in ID, potentially due to targeted interventions such as vaccinations and treatments that are effectively captured by higher spending levels.^15^ Conversely, the minimal and non-significant impacts on RMNCH and NCDs suggest that simply increasing expenditure may not be sufficient without strategic allocation towards these areas.^16^

 The analysis also found that Education plays a crucial role in improving health outcomes. The significant positive association between the primary completion rate and improved health outcomes in RMNCH, NCDs, and IDs supports existing theories that education acts as a social determinant of health. Educated individuals may have better access to information and resources, leading to healthier lifestyle choices and increased utilization of healthcare services.^17^ The lack of a significant association with SC outcomes might be attributed to the complex needs of social care that extend beyond the benefits conferred by primary education alone.

 Our findings show that demographic changes reveal a dual burden, with an aging population negatively impacting RMNCH and IDs but positively influencing NCDs and SC outcomes, while a growing working-age population is linked to higher infectious disease transmission. While there is a significant negative impact on RMNCH outcomes and a non-significant negative impact on IDs, there is a positive effect on NCDs and SC outcomes.^5^ This could reflect the increased healthcare needs and resource allocation towards managing chronic diseases and providing SC for the elderly.^18^ The growth in the working-age population showing a positive correlation with IDs but not with other health outcomes might indicate increased social interactions and mobility, leading to higher transmission rates of IDs.^19^

 It was observed that access to basic sanitation services significantly boosts health outcomes across RMNCH, NCDs, and IDs, corroborating the notion that sanitation is crucial for preventing disease spread and improving overall health.^20^ The stronger effect on IDs can be directly linked to reduced transmission of waterborne and sanitation-related infections.^21^ The smaller impact on SC may suggest that factors other than basic sanitation predominantly influence these outcomes.

 A strong positive correlation between life expectancy and better health outcomes across all categories strongly affirms the utility of life expectancy as a general indicator of public health and healthcare effectiveness.^22^ Each additional year in life expectancy correlates with significant improvements in health outcomes, reflecting broader health system achievements.

 Finally, the lack of measurable impact from measles cases on health outcomes highlights the success of vaccination efforts and suggests that measles is no longer a significant indicator of broader health metrics in well-controlled settings. The null effects of measles cases on health outcomes may suggest successful control and vaccination efforts that limit the broader health impact of measles outbreaks. ^23^ Alternatively, it may indicate that measles does not serve as a significant indicator of general health outcomes in the presence of effective vaccination programs.

## Limitations of the study

 While the study provides valuable insights into the relationship between PHE and UHC, several limitations must be acknowledged. First, the analysis relies on secondary data from sources such as the World Bank and the World Health Organization, which may contain inconsistencies or gaps, particularly in low-income countries with weaker data reporting systems. Second, the study’s reliance on proxy measures, such as OOP payments for financial risk protection, may not fully capture the complexities of healthcare financing and access. Third, the use of a fixed and random effects model, though robust, does not account for potential endogeneity or reverse causality between health outcomes and expenditure. Additionally, while the decomposition of UHC into specific indices (e.g., RMNCH, ID, NCD, and SC) allows for granular analysis, it may overlook broader systemic factors influencing health outcomes, such as governance, cultural practices, or geopolitical constraints. Finally, the study focuses on average trends and does not account for regional or subnational disparities within countries, which can significantly impact health coverage and outcomes.

## Conclusion and Policy Implications

 The results of the fixed effect models used in our analysis have illuminated significant drivers of health outcomes in the categories of RMNCH, NCDs, IDs, and SC. Key findings from the study suggest that strategic public health investments and policies could significantly enhance health outcomes across these categories. Our findings strongly support the need for increased health expenditure, particularly in the area of ID, where expenditure is directly correlated with improved health outcomes. Policy recommendations should focus on increasing and strategically allocating health expenditure to optimize the impact on health outcomes, especially in areas like ID that show a high responsiveness to funding.

 Education emerges as another critical determinant of health, with the primary completion rate significantly influencing health outcomes in RMNCH, NCDs, and IDs. Policies aimed at improving educational attainment could indirectly contribute to better health outcomes, emphasizing the need for integrated approaches that consider education as a part of public health strategies. The demographic shifts, notably the aging population, require targeted health policies to accommodate an increasing need for NCD and SC management, thus ensuring resources are adequately directed towards these increasingly pressing areas.

 Furthermore, our analysis underscores the importance of basic sanitation services, which have shown significant positive impacts on health outcomes. Policies enhancing access to sanitation and clean water can lead to broad public health benefits, particularly in combating IDs. It is recommended that public health policies integrate environmental health improvements to reduce disease transmission and improve overall health. Given the comprehensive and robust nature of the findings across different health outcomes and variables, policy implementations should prioritize multi-sectoral approaches that recognize the interdependencies of health determinants and strategically utilize resources to maximize public health gains.

## Competing Interests

 The authors declare no conflict of interest.

## Data Availability Statement

 All data is available on WDI and WHO website. Stata data can be provided on request by Khalilullah.buic@bahria.edu.pk.

## Ethical Approval

 The study uses published secondary data and does not any human subjects therefore no ethical approvals are required.

## Supplementary Files


Supplementary file 1. List of countries used in analysis.

Supplementary file 2. Data sources

Supplementary file 3. Hausman text

Supplementary file 4. Breusch-Pagan test

Supplementary file 5. Variance Inflation Factors for Multicollinearity.

Supplementary file 6. Random effects estimation results for health outcomes by UHC component
