# Supplementary material for: Impact of health expenditure on universal health coverage (UHC) (composite index): Global evidence
Source: Health Promot Perspect. 2025 Nov 4;15(3):268–77. doi: 10.34172/hpp.025.43192 (PMC12680523; doi:10.34172/hpp.025.43192)
Supplement: Supplementary file 1 — List of countries used in analysis. [file hpp-15-268-s001.pdf]

Khan et al, **Health Promotion Perspectives.** 2025;15(2):268-S1
doi: 10.34172/hpp.025.43192
https://hpp.tbzmed.ac.ir

Supplementary file 1

List of Countries Used in Analysis

Country Name			
Afghanistan	Equatorial Guinea	Mali	Sri Lanka
Albania	Eritrea	Malta	St. Kitts and Nevis
Algeria	Estonia	Marshall Islands	St. Lucia
Angola	Eswatini	Mauritania	St. Vincent and the Grenadines
Antigua and Barbuda	Ethiopia	Mauritius	Sudan
Argentina	Fiji	Mexico	Suriname
Armenia	Finland	Micronesia, Fed. Sts.	Sweden
Austria	Gabon	Moldova	Switzerland
Azerbaijan	Gambia, The	Mongolia	Syrian Arab Republic
Bahamas, The	Georgia	Montenegro	Tajikistan
Bahrain	Germany	Morocco	Tanzania
Bangladesh	Ghana	Mozambique	Thailand
Barbados	Greece	Myanmar	Timor-Leste
Belarus	Grenada	Namibia	Togo
Belize	Guatemala	Nauru	Tonga
Benin	Guinea	Nepal	Trinidad and Tobago
Bhutan	Guinea-Bissau	Nicaragua	Tunisia
Bolivia	Guyana	Niger	Turkiye
Bosnia and Herzegovina	Haiti	Nigeria	Turkmenistan
Botswana	Honduras	North Macedonia	Tuvalu
Brunei Darussalam	Hungary	Norway	Uganda
Bulgaria	Iceland	Oman	Ukraine
Burkina Faso	India	Pakistan	United Arab Emirates
Burundi	Indonesia	Panama	United Kingdom
Cabo Verde	Iran, Islamic Rep.	Papua New Guinea	United States
Cambodia	Iraq	Paraguay	Uruguay
Cameroon	Ireland	Peru	Uzbekistan
Central African Republic	Israel	Philippines	Vanuatu
Chad	Italy	Poland	Viet Nam

Chile	Jamaica	Portugal	Yemen, Rep.
China	Jordan	Qatar	Zambia
Colombia	Kazakhstan	Romania	Zimbabwe
Comoros	Kenya	Russian Federation	
Congo, Dem. Rep.	Kiribati	Rwanda	
Congo, Rep.	Korea, Rep.	Samoa	
Costa Rica	Kuwait	Sao Tome and Principe	
Cote d'Ivoire	Kyrgyz Republic	Saudi Arabia	
Croatia	Lao PDR	Senegal	
Cuba	Latvia	Serbia	
Cyprus	Lebanon	Seychelles	
Czechia	Lesotho	Sierra Leone	
Denmark	Liberia	Singapore	
Djibouti	Lithuania	Slovak Republic	
Dominica	Luxembourg	Slovenia	
Dominican Republic	Madagascar	Solomon Islands	
Ecuador	Malawi	South Africa	
Egypt, Arab Rep.	Malaysia	South Sudan	
El Salvador	Maldives	Spain	
