# Supplementary material for: Impact of health expenditure on universal health coverage (UHC) (composite index): Global evidence
Source: Health Promot Perspect. 2025 Nov 4;15(3):268–77. doi: 10.34172/hpp.025.43192 (PMC12680523; doi:10.34172/hpp.025.43192)
Supplement: Supplementary file 2 — Data sources [file hpp-15-268-s002.pdf]

Khan et al, **Health Promotion Perspectives.** 2025;15(2):268-S2
doi: 10.34172/hpp.025.43192
https://hpp.tbzmed.ac.ir

Supplementary file 2. Data sources

Variables Name	Data Source	Variable Type
**UHC Index**	World Development Indicators (WDI) - https://data.worldbank.org/	Dependent Variable
**RMNCH**	https://www.who.int/data/gho/indicator-metadata-registry/imr-details/4834	Dependent Variable
**ID**	https://www.who.int/data/gho/indicator-metadata-registry/imr-details/4834	Dependent Variable
**NCD (Non Communicable Diseases by type of diseases and sex)**	https://www.who.int/data/gho/indicator-metadata-registry/imr-details/4834	Dependent Variable
**Service Capacity and access**	https://www.who.int/data/gho/indicator-metadata-registry/imr-details/4834	Dependent Variable
**Life Expectancy at Birth (total years)**	World Development Indicators (WDI) - https://data.worldbank.org/	Confounding/ Controls
**Mortality rate, infant (per 1,000 live births)**	World Development Indicators (WDI) - https://data.worldbank.org/	Confounding/Controls
**Domestic general health expenditure per capita (current US$)**	World Development Indicators (WDI) - https://data.worldbank.org/	Independent Variable
**GDP per capita (current US%)**	World Development Indicators (WDI) - https://data.worldbank.org/	Confounding/Controls
**Primary completion rate, total (% of relevant age group)**	World Development Indicators (WDI) - https://data.worldbank.org/	Confounding/Controls
**Population aged 65 and above (% of total population)**	World Development Indicators (WDI) - https://data.worldbank.org/	Confounding/Controls
**Population aged between 15 and 64 (% of total population)**	World Development Indicators (WDI) - https://data.worldbank.org/	Confounding/Controls
**People using at least basic drinking water services (% of population)**	World Development Indicators (WDI) - https://data.worldbank.org/	Confounding/Controls
**People using at least basic sanitation services (% of population)**	World Development Indicators (WDI) - https://data.worldbank.org/	Confounding/Controls

**Measles (number of reported cases)**	https://www.who.int/data/gho/data/indicators/indicator-details/GHO/measles---number-of-reported-cases	Confounding/Controls
**Out Of Pocket Expenditure per capita (current US$)**	World Development Indicators (WDI) - https://data.worldbank.org/	Independent Variable
**GDP Constant (US$)**	World Development Indicators (WDI) - https://data.worldbank.org/	Confounding/Controls
