# Supplementary material for: Impact of health expenditure on universal health coverage (UHC) (composite index): Global evidence
Source: Health Promot Perspect. 2025 Nov 4;15(3):268–77. doi: 10.34172/hpp.025.43192 (PMC12680523; doi:10.34172/hpp.025.43192)
Supplement: Supplementary file 3 — Hausman text [file hpp-15-268-s003.pdf]

Khan et al, **Health Promotion Perspectives.** 2025;15(2):268-S3
doi: 10.34172/hpp.025.43192
https://hpp.tbzmed.ac.ir

Supplementary file 3. Hausman text

Hausman (1978) specification test

	Coef.
Chi-square test value	382.536
P-value	0.00
