# Supplementary material for: Impact of health expenditure on universal health coverage (UHC) (composite index): Global evidence
Source: Health Promot Perspect. 2025 Nov 4;15(3):268–77. doi: 10.34172/hpp.025.43192 (PMC12680523; doi:10.34172/hpp.025.43192)
Supplement: Supplementary file 4 — Breusch-Pagan test [file hpp-15-268-s004.pdf]

Khan et al, **Health Promotion Perspectives.** 2025;15(2):268-S4
doi: 10.34172/hpp.025.43192
https://hpp.tbzmed.ac.ir

Supplementary file 4. Breusch-Pagan test

Breusch-Pagan / Cook-Weisberg test for heteroskedasticity	
Ho: Constant variance	
Variables: fitted values of UHC index	
chi2(1) = 55.74	
Prob > chi2 = 0.0000	
