# Supplementary material for: Impact of health expenditure on universal health coverage (UHC) (composite index): Global evidence
Source: Health Promot Perspect. 2025 Nov 4;15(3):268–77. doi: 10.34172/hpp.025.43192 (PMC12680523; doi:10.34172/hpp.025.43192)
Supplement: Supplementary file 5 — Variance Inflation Factors for Multicollinearity. [file hpp-15-268-s005.pdf]

Khan et al, **Health Promotion Perspectives.** 2025;15(2):268-S5
doi: 10.34172/hpp.025.43192
https://hpp.tbzmed.ac.ir

Supplementary file 5. Variance inflation factors for multicollinearity

Variance inflation factor

	VIF	1/VIF
Life Expectancy at Birth (total years)	5.93	.169
People using at least basic drinking water services (% of population)	5.715	.175
People using at least basic sanitation services (% of population)	5.135	.195
Domestic general health expenditure per capita (current US$)	4.835	.207
Population aged between 15 and 64 (% of total population)	3.539	.283
Primary completion rate, total (% of relevant age group)	3.099	.323
Population aged 65 and above (% of total population)	2.562	.39
Measles (number of reported cases)	1.235	.81
Mean VIF	4.006	.

Table S1. Random Effects Estimation Results: Impact of Health Expenditure on UHC and Income-Level Interaction

	Hypothesis 1	Hypothesis 2	Nonlinearity
Variables	Coefficient/Robust Standard Errors	Coefficient/Robust Standard Errors	Coefficient/Robust Standard Errors
DGG health expenditure per capita	0.0719***	0.0778***	0.1381***
	(0.0125)	(0.0123)	(0.0340)
(DGG health expenditure per capita)^2			-0.0078**
			(0.0036)
Primary completion rate	0.0032***	0.0029***	0.0025***
	(0.0007)	(0.0007)	(0.0007)
Population ages 65 and above (% of total population)	-0.0075**	-0.0013	0.0011
	(0.0029)	(0.0026)	(0.0025)

Population ages 15-64 (% of total population)	0.0014	0.0017	0.0028
	(0.0028)	(0.0028)	(0.0028)
Basic drinking water services (% of population)	0.0026	0.0029	0.0022
	(0.0019)	(0.0018)	(0.0019)
Basic sanitation services (% of population)	0.0009	0.0006	0.0005
	(0.0012)	(0.0012)	(0.0012)
Measles (number of reported cases)	-0.0000	-0.0000	-0.0000
	(0.0000)	(0.0000)	(0.0000)
Life expectancy at birth, total (years)	0.0249***	0.0242***	0.0246***
	(0.0041)	(0.0040)	(0.0039)
HigherIncome x Domestic Expenditure		-0.0000***	-0.0000
		(0.0000)	(0.0000)
LowerMidIncome x Domestic Expenditure		0.0008**	0.0008**
		(0.0003)	(0.0002)
LowerIncome x Domestic Expenditure		0.0087**	0.0072*
		(0.0043)	(0.0040)
Constant	1.3284***	1.3069***	1.2012***
	(0.2381)	(0.2243)	(0.2173)
Overall Rsquare	.8472	.8533	.8560
No. of Obs	696	696	696
No. of Groups	169	169	169
Reference Dummy is Middle income Country	----------------	---------------	------------------

* p<.1, ** p<.05, *** p<.001

The results from Equation 4a-c estimations using random effects models provide insights into the factors affecting UHC across different income levels of countries and test for non-linear effects of health expenditure on UHC.

Hypothesis 1 (Impact of Health Expenditure on UHC):

- **DGG health expenditure per capita** has a significant positive effect on UHC (Coefficient = 0.0719, $p < 0.001$), indicating that as health expenditure increases, UHC also tends to improve. This strong association suggests that higher per capita spending in health leads to better coverage outcomes.

Hypothesis 2 (Impact of Income Level on UHC):

- **DGG health expenditure per capita** continues to show a significant positive effect (Coefficient = 0.0778, $p < 0.001$), slightly stronger than in Hypothesis 1.
- Interactions between income levels and domestic expenditure show mixed results:
  - **HigherIncome x Domestic Expenditure**: The coefficient is negative and significant (Coefficient = -0.0000, $p < 0.001$), indicating that higher income countries may see diminishing returns from additional health spending.
  - **LowerMidIncome x Domestic Expenditure**: Shows a positive relationship (Coefficient = 0.0008, $p < 0.05$), suggesting that lower-middle-income countries benefit moderately from increases in health spending.
  - **LowerIncome x Domestic Expenditure**: Also positive (Coefficient = 0.0087, $p < 0.05$), implying that low-income countries benefit significantly from increased health expenditure, possibly because of the larger gaps in health coverage that need to be addressed.

Nonlinear Hypothesis (Non-linear Relationship between Health Expenditure and UHC):

- **DGG health expenditure per capita** shows a much stronger positive effect on UHC in the non-linear model (Coefficient = 0.1381, $p < 0.001$).
- The squared term of health expenditure (**(DGG health expenditure per capita)^2**) is negative and significant (Coefficient = -0.0078, $p < 0.05$), indicating a diminishing return effect; as health spending increases, the incremental benefits on UHC decrease.
