# Supplementary material for: Impact of health expenditure on universal health coverage (UHC) (composite index): Global evidence
Source: Health Promot Perspect. 2025 Nov 4;15(3):268–77. doi: 10.34172/hpp.025.43192 (PMC12680523; doi:10.34172/hpp.025.43192)
Supplement: Supplementary file 6 — Random effects estimation results for health outcomes by UHC component [file hpp-15-268-s006.pdf]

Khan et al, **Health Promotion Perspectives.** 2025;15(2):268-S6
doi: 10.34172/hpp.025.43192
https://hpp.tbzmed.ac.ir

Supplementary file 6. Random effects estimation results for health outcomes by UHC component

Variables	Decomposition			
	RMNCH	NCD	ID	SC
	Coefficient/Robust Standard Errors	Coefficient/Robust Standard Errors	Coefficient/Robust Standard Errors	Coeffi[illegible] Stand[illegible]
DGG health expenditure per capita	0.0289**	0.0175	0.1807***	-0.005[illegible]
	(0.0114)	(0.0108)	(0.0294)	(0.014[illegible]
Primary completion rate	0.0043***	0.0014**	0.0064**	0.000[illegible]
	(0.0006)	(0.0006)	(0.0025)	(0.001[illegible]
Population ages 65 and above (% of total population)	-0.0065**	0.0089***	0.0256***	0.020[illegible]
	(0.0028)	(0.0026)	(0.0073)	(0.003[illegible]
Population ages 15-64 (% of total population)	-0.0037*	0.0015	-0.0082	0.006[illegible]
	(0.0022)	(0.0017)	(0.0089)	(0.003[illegible]
Basic drinking water services (% of population)	0.0007	0.0007	-0.0006	0.007[illegible]
	(0.0023)	(0.0019)	(0.0049)	(0.002[illegible]
Basic sanitation services (% of population)	0.0012	0.0018	-0.002	0.006[illegible]
	(0.0012)	(0.0017)	(0.003)	(0.001[illegible]
Measles (number of reported cases)	<0.0001	<0.0001	<0.0001	<0.00[illegible]
	(<0.0001)	(<0.0001)	(<0.0001)	(<0.00[illegible]
Life expectancy at birth, total (years)	0.0149***	0.0129***	0.0542***	0.000[illegible]
	(0.0042)	-0.0028	-0.0092	-0.003[illegible]
Constant	2.8206***	2.5557***	-0.5334	2.260[illegible]
	(0.2794)	(0.163)	(0.514)	(0.268[illegible]
Overall R-square	0.702342	0.2805094	0.58547	0.826[illegible]
No. of Observations	696	696	696	696
No. of Groups	169	169	169	169

The results from Table 3's Random Effect Estimation provide insights into various health-related outcomes influenced by demographic and socioeconomic factors. Starting with DGG health expenditure per capita, the results indicate a significant positive effect on RMNCH and ID, with coefficients of 0.0289 (significant at the 5% level) and 0.1807 (significant at the 1% level) respectively, highlighting the importance of health spending in improving health outcomes in these areas. However, the effect on NCD and SC is either not significant or slightly negative, suggesting that health expenditure does not impact these areas in the same way.

Further examination of factors such as the primary completion rate and population demographics reveals additional impacts on health outcomes. A higher primary completion rate significantly improves RMNCH, NCD, and ID outcomes, suggesting that education plays a crucial role in enhancing health. Similarly, the percentage of the population aged 65 and above shows varied effects; it negatively impacts RMNCH and ID outcomes but positively affects NCD and SC outcomes, indicating that an older population may require different health resources. On the other hand, basic sanitation services show a positive effect on SC outcomes, marked by a significant coefficient, reinforcing the role of sanitation in improving SC health metrics.

Lastly, the life expectancy variable significantly improves health outcomes across RMNCH, NCD, and ID but not SC, underscoring life expectancy as a critical indicator of overall health quality. The overall R-squared values for these models are notably high for RMNCH and SC, suggesting a good fit for the data in explaining the variability of these outcomes. In contrast, the fit is weaker for NCD outcomes, indicating that other unexplored variables might play a role in these models. These results underscore the complex interplay of various socioeconomic factors in shaping public health outcomes and the need for tailored strategies addressing different health domains.
